# Hippocampal Blood Flow Is Increased After 20 min of Moderate-Intensity Exercise

**DOI:** 10.1093/cercor/bhz104

**Published:** 2019-06-19

**Authors:** J J Steventon, C Foster, H Furby, D Helme, R G Wise, K Murphy

**Affiliations:** 1 Neuroscience and Mental Health Research Institute, School of Medicine, Cardiff University, Cardiff, CF24 4HQ, UK; 2 School of Physics and Astronomy, The Parade, Cardiff University, Cardiff, CF24 3AA, UK; 3 Cardiff University Brain Research Imaging Centre, School of Psychology, Cardiff University, Cardiff, CF24 4HQ, UK; 4 Institute of Neurology, University College London, London, WC1B 5EH, UK; 5 Department of Anaesthetics and Intensive Care Medicine, School of Medicine, Cardiff University, Cardiff, CF14 4XN, UK

**Keywords:** cerebral blood flow, cerebrovascular reactivity, hippocampus, MRI, plasticity

## Abstract

Long-term exercise interventions have been shown to be a potent trigger for both neurogenesis and vascular plasticity. However, little is known about the underlying temporal dynamics and specifically when exercise-induced vascular adaptations first occur, which is vital for therapeutic applications. In this study, we investigated whether a single session of moderate-intensity exercise was sufficient to induce changes in the cerebral vasculature. We employed arterial spin labeling magnetic resonance imaging to measure global and regional cerebral blood flow (CBF) before and after 20 min of cycling. The blood vessels’ ability to dilate, measured by cerebrovascular reactivity (CVR) to CO_2_ inhalation, was measured at baseline and 25-min postexercise. Our data showed that CBF was selectively increased by 10–12% in the hippocampus 15, 40, and 60 min after exercise cessation, whereas CVR to CO_2_ was unchanged in all regions. The absence of a corresponding change in hippocampal CVR suggests that the immediate and transient hippocampal adaptations observed after exercise are not driven by a mechanical vascular change and more likely represents an adaptive metabolic change, providing a framework for exploring the therapeutic potential of exercise-induced plasticity (neural, vascular, or both) in clinical and aged populations.

## Introduction

There is accumulating evidence that exercise can produce functional improvements in a range of neurological conditions such as stroke (Tang et al. 2014; Robertson et al. 2015), Parkinson’s disease (Mehrholz et al. 2016), multiple sclerosis (Snook and Motl 2009; Tallner et al. 2016), and Alzheimer’s disease (Hoffmann et al. 2016). However, the neurobiological mechanisms associated with this low-cost therapeutic intervention are not well understood.

Following long-term exercise training, the most robust and extensively researched pathway specifically for cognitive gains is increased neurogenesis in the hippocampus in the human and rodent brain (Cotman and Berchtold 2002; van Praag et al. 2005; Pereira et al. 2007; Erickson et al. 2011). Nonetheless, there is increasing evidence that exercise also induces cerebrovascular plasticity (Lopez-Lopez et al. 2004; Kojda and Hambrecht 2005; Holschneider et al. 2007; Pereira et al. 2007; Burdette et al. 2010; Rhyu et al. 2010). Neurogenesis and angiogenesis are tightly coupled processes (Chopp et al. 2007), and in the animal literature, efforts have been made to distinguish between exercise-induced neurogenic and angiogenic processes. For example, using in vivo magnetic resonance imaging (MRI), a 2-week long exercise intervention in mice selectively increased cerebral blood volume in the dentate gyrus, which was found to correlate with a postmortem marker of neurogenesis (Pereira et al. 2007), suggesting that the exercise-induced changes in cerebral blood volume were accounted for by neurogenesis. Similarly, evidence suggests that observed changes in cerebral blood flow (CBF) in the hippocampus during exercise are neuronally mediated. For example, in a laser Doppler flowmetry study in rats, Nishijima and Soya (2006) demonstrated that hippocampal blood flow was increased during mild treadmill running (10 m/min for 20 min), and this effect was entirely suppressed by the infusion of tetrodotoxin (Nishijima and Soya 2006)—a sodium channel blocker, which does not affect microvascular tone.

In order to determine the source of exercise-induced changes in blood flow, it is necessary to account for exercise-induced systemic changes, which can independently contribute to observed alterations in the cerebrovasculature. For example, during exercise, intensity-dependent changes in various physiological factors contribute to changes observed in CBF (Smith and Ainslie 2017). Cerebral blood velocity in the middle cerebral artery and CBF in the internal carotid artery increase linearly at exercise intensities up to 60% of maximum oxygen consumption and then progressively decrease toward baseline during heavy exercise (Ogoh and Ainslie 2009; Sato et al. 2011; Smith and Ainslie 2017) despite continued increasing neuronal activation (Ide and Secher 2000; Ogoh and Ainslie 2009; Sato et al. 2011). This temporal pattern of CBF during exercise is mediated by changes in the arterial partial pressure of carbon dioxide (PaCO_2_), which has a major influence on arteriolar diameter (Thomas et al. 1989; Ide and Secher 2000; Ratanakorn et al. 2001).

Despite the accumulating evidence base for cerebrovascular changes in the hippocampus during exercise and following long-term exercise interventions, only a few studies have considered the period immediately after exercise cessation. Previous work using resting-state functional MRI found that following 30 min of aerobic exercise, the integration of attention, executive control, and hippocampal networks was increased (Weng et al. 2017). Only 2 human experiments have examined acute changes in cerebral perfusion in healthy control participants after exercise using arterial spin labeling (ASL) perfusion MRI, which is sensitive to the signal from the rapid exchange of magnetically labeled blood water molecules in the tissue capillary bed. In a small ASL-MRI study (*n = 5*), Smith et al. (2010) reported that global perfusion was significantly elevated up to 30 min following a 30-min bout of moderate intensity exercise. In contrast, in 16 volunteers, Macintosh et al. (2014) found that hippocampal perfusion was significantly reduced 10 min and 40 min after 20 min of cycling at 70% of the age-predicted maximal heart rate (HR). However, the large-scale effects of exercise on the cardio-pulmonary systems (Piepoli et al. 1993; Halliwill et al. 1996a, 1996b) were not accounted for in the latter study while the former study was limited by sample size, which may explain the contradictory results.

In this study, we sought to extend the work from previous studies and investigate the time course of exercise-induced cerebral adaptations, while accounting for the systemic changes (e.g., changes in PCO_2_ and blood pressure), which also occur following exercise and may contribute to perfusion regulation. Accordingly, along with measuring resting perfusion we also directly manipulated PaCO_2_ and assessed changes in cerebrovascular reactivity (CVR) before and after exercise. Whereas neurogenic, metabolic, autoregulatory, and systemic factors contribute to perfusion regulation (Smith and Ainslie 2017), CVR is related to the mechanical properties of arterial vessels and provides a dynamic measure of the vessels ability to modulate vascular tone in response to a vasoactive stimuli, which may be intrinsically altered by exercise. Measuring both the change in resting perfusion and CVR following exercise provides unique insight into blood flow regulation and neural activity after exercise. Specifically, we examined the hypotheses that localized hippocampal perfusion is elevated immediately after exercise cessation and sought to assess whether this was driven by changes in vascular function using a hypercapnia manipulation.

## Materials and Methods

**Figure 1:**
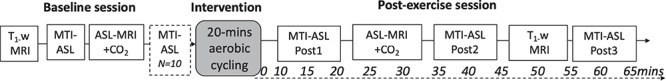
Study design. MRI measures were recorded up to 65 min after exercise cessation. MTI-ASL means multi-inversion time ASL MRI. ASL-MRI + CO2, single TI ASL MRI with hypercapnia challenge. T_1−_w, T_1_-weighted structural MRI scan acquired for image registration purposes. Cycling was performed on an upright ergometer. The dashed box in the baseline session represents the reliability MTI-ASL scan performed in a subset of participants.

The study design is detailed in Figure 1. Supporting data, analysis code, and how to access the supporting data are available at http://doi.org/10.17035/d.2019.0070728670.

### Participants

Previous work has shown a very large effect size (dz = 5.57) of long-term exercise on cerebral blood volume, with the difference between the pre-exercise and postexercise session larger than 3 standard deviations (Pereira et al. 2007). We calculated that with an effect size of 0.9 (given the acute nature of the intervention) and 95% power, we would require a minimum of 28 participants. Accordingly, 32 healthy participants (17 males and 15 females) were recruited in total; demographic features are shown in Table 1. The inclusion criteria included 18–55 years old, non-smoking, non-hypertensive, and free from known neurological, respiratory, and cardiovascular diseases. All participants were asked to refrain from vigorous physical activity and alcohol consumption for at least 12 h prior to experimentation. All participants gave informed consent at each session and underwent a screening interview, which included additional exclusion criteria for contraindications, exercise, and respiratory gas modulation. All procedures were granted ethical approval from Cardiff University School of Psychology Ethics Committee (EC.15.01.13.4064).

**Table 1:** Subject demographics

*N*	32
Gender (M: males)	17 M
Age (years)	33.2 ± 1.8 (19.9–52.1)
BMI (kg/m^2^)	24.8 ± 0.5 (20–32)
SBP (mmHg)	121.5 ± 1.8 (103–141)
DBP (mmHg)	70.6 ± 1.7 (56–94)
VO_2_ max (ml/kg/min)	38.6 ± 1.7 (25–61)

Values are mean ± SEM, range in parentheses. BMI means body mass index; SBP, systolic blood pressure; DBP, diastolic blood pressure. Note that CVR was measured in a subset of participants in cohort 1 (*n* = 16; 26.2 ± 3.4 years old; 7 males)*.*

### Exercise Intervention

Participants underwent 20 min of moderate-intensity aerobic cycling on an upright ergometer (Lode ergometer; Lode). To ensure a moderate-intensity aerobic intervention, a prescribed intensity of 50–70% of the maximal HR reserve (HRR) was determined using the Karvonen formula (American College of Sports Medicine et al. 2012) with maximum HR calculated as (220 age).

$$ \textrm{HRR}=\mathrm{resting}\ \mathrm{HR}+\left(\mathrm{target}\ \mathrm{HR}\%\times \left[\mathrm{MaxHR}-\mathrm{Resting}\ \mathrm{HR}\right]\right)$$

Participants first rested on the upright ergometer for 2 min before commencing a 2-min warm up at 25 W. Following this, the resistance was adjusted to target the HRR and was manually adjusted at 2-min intervals to ensure HR was maintained with the HRR zone. During upright rest, the warm-up and warm-down period, and at 3-min intervals during exercise, whole blood capillary lactate concentration was collected from the left earlobe, blood pressure was measured, and self-report ratings of perceived exertion were recorded using a 10-point Borg scale(Borg 1974). After 20 min, participants completed a 2-min warm down at 25 W. Immediately after this, participants returned to the MR scanner.

### Perfusion Acquisition

MRI data were acquired on a 3 T whole-body MRI system (GE Excite HDx) equipped with a body transmit and 8-channel receive head coil. To measure CBF, multi-inversion time pulsed ASL (PASL) data were acquired using a PICORE (proximal inversion with a control for off-resonance effects) sequence (Wong et al. 1997) with a dual-echo gradient echo readout. Data were acquired at 8 inversion times (time to inversion (TI)_1–8_ = 400, 500, 600, 700, 1100, 1400, 1700, 2000 ms). A quantitative imaging of perfusion with a single subtraction (Wong et al. 1998) cut-off of the label was applied at 700 ms for TIs > 700, which meant that short (<700 ms) and long inversion times (>700 ms) were acquired in separate runs; 16 and 8 tag-control pairs were acquired for each short and long inversion times, respectively. A variable repetition time (TR) was used for efficiency (echo time (TE) = 2.7 ms, 15 slices [7 mm thick +1.5 mm gap], slice delay = 52 ms, FOV = 198 mm, 64 × 64 matrix = ~ 3.1 mm^2^ in-plane resolution, 20 cm labeling thickness with 1 cm gap between the most proximal imaging slice, flip angle = 90°).

To estimate the equilibrium magnetization (M_0_) of arterial blood, a single echo spiral *k*-space scan was acquired with the same parameters as above, minus the ASL tag preparation.

For registration purposes, a 3D T_1_-weighted fast spoiled gradient echo sequence was acquired at baseline and in the postexercise scan session (256 × 256, slice thickness = 1 mm giving a resolution of 1 mm^3^, TR/TE of 7.90/3.0 ms). The 3 postexercise perfusion scans were acquired at the same time postexercise cessation in all participants (see Fig. 1 for timings).

In a subset of participants (*n* = 10), the multi-TI PASL sequence acquisition was repeated at the end of the baseline scan session to measure the reliability of baseline perfusion (dashed box in Fig. 1).

### Hypercapnic Cerebral Vascular Reactivity Acquisition

To measure CVR in response to a hypercapnic challenge, a single TI dual-echo pulsed ASL was used to acquire perfusion and blood oxygen level-dependent (BOLD) time series simultaneously (15 slices, matrix = 64 × 64, TE_1_ = 2.7 ms, TE_2_ = 29 ms, TR = 2.4 s, TI = 1.5 s, FOV = 19.8 cm, flip angle = 90°, slice thickness/gap 7/1.5 mm, 255 volumes) with a dual-echo gradient echo readout and spiral acquisition of *k*-space in a subset of participants (*n* = 16; 26.2 ± 3.4 years old; [age range = 20–30], 7 males). During this scan, participants breathed through a tight-fitting face mask covering the nose and mouth (Quadralite; Intersurgical). Humidified gases were delivered from gas cylinders connected to an in-house manually operated system of flow meters. A moderate degree of hypercapnia (+7–8 mmHg end-tidal CO_2_) was induced in a block-design fashion (3 blocks of normocapnia, 2 blocks of hypercapnia, 120 s each). During the normocapnic period, participants breathed medical air (20.9% O_2_ balance N_2_) with a flow rate of 30 L/min. Hypercapnia was manually controlled by mixing medical air with 5% CO_2_ until partial pressure of end-tidal respiratory carbon dioxide (PETCO_2_) was raised by 7–8 mmHg above individual participant’s baseline.

### Cardiorespiratory Measures

Pulse waveforms and oxygen saturation were recorded (Medrad) and blood pressure measurements were collected using an arm cuff before and after each scan (OMRON). Expired gas content was recorded (AEI Technologies) and sampled at 500 Hz (CED) to obtain measures of P_ET_CO_2_. A respiratory belt was placed just below the ribs to monitor ventilation.

### CBF quantification

Data were analyzed in AFNI version 16.2.18 (Cox 1996) and using the FMRIB Software Library version 5.0 (FSL, http://fsl.fmrib.ox.ac.uk; Jenkinson et al. 2012). To correct for motion within each condition, time-series images were spatially registered using 3dAllineate within AFNI with a local Pearson correlation cost function and 6-parameter warp with a 1-pass refining strategy.

Brain extraction was performed on the first image of the raw motion corrected series and the cerebrospinal fluid (CSF) calibration image with the FSL brain extraction tool. The CSF image was registered to the perfusion image, and a threshold mask of the lateral ventricles was used to calculate the M0_CSF,_ following the procedures described by Warnert et al. (2015). The M0 for arterial blood was then calculated according to methods described by Wong et al. (1998) with CSF as a reference. Perfusion quantification was performed on a voxel-by-voxel basis using a 2-compartment model (Chappell et al. 2010) and employing partial volume correction (Chappell et al. 2011) within the BASIL toolkit (Groves et al. 2009) within the FMRIB Software Library.

To avoid voxels affected by poor SNR in white matter and to exclude nonphysiological values, CBF images were masked for partial volume gray matter values greater than 40% and estimated values of perfusion within the range (0200) ml/100 g/min. Global gray matter CBF and hippocampal CBF were calculated for the 4 perfusion images (baseline, post-1, post-2, and post-3) in line with our hypotheses. In addition, in order to determine if any effects were specific to the hippocampus, we also measured regional changes in the thalamus (a key hub for the motor system) and in 3 cortical regions of interest (ROIs)—the middle frontal gyrus, postcentral gyrus, and precentral gyrus, selected based on the exercise intervention neuroimaging literature (Pereira et al. 2007; Smith et al. 2010; MacIntosh et al. 2014). ROIs were segmented using masks obtained from Harvard-Oxford cortical and subcortical probabilistic atlases provided within the FMRIB Software Library. Masks were then registered to participant perfusion space, and the median perfusion value was calculated for each ROI.

### CVR Data Analysis

Image time series were motion corrected using 3dvolreg (Cox 1996) and brain extracted using 3dAutomask within AFNI (Cox 1996). Interpolated surround subtraction was performed on the ASL tag-control image time series to yield a perfusion-weighted time series from the first echo, and a BOLD-weighted time series was calculated from the second echo with surround averaging of the tag and control pairs. Using a similar M0 calculation as described above, quantified perfusion values were calculated using a single compartment model (Wong et al. 1998).

The PETCO_2_ trace was convolved with a single hemodynamic response function and fitted to the perfusion and BOLD image time series in each subject, using 3dDeconvolve (Cox 1996) to obtain CVR, measured as %CBF or %BOLD signal change per mmHg PETCO_2_ change. To avoid voxels affected by poor SNR and/or a poor model fit, CVR images were masked based on the R^2^ of the fit and CBF CVR masks included only positive integers. Median gray matter values were then calculated to assess global CVR while regional changes in CVR were assessed in the same ROIs as detailed above.

### Baseline Fitness Test

The maximum volume of oxygen consumption (VO2max), the gold standard measure of exercise-associated aerobic fitness (Wagner 1996), was measured by a graded exercise test on an upright cycle ergometer (Steventon et al. 2018) (1000 W; Cranlea Human Performance Ltd) on a separate day within a month of the MRI session in all participants. Following a 2-min rest period and a 2-min warm-up period at 0 W, participants began exercising at 50 W with the work rate continually increased by 25 W every 2 min until VO2 max criteria (respiratory quotient > 1.1, within 10 beats/min of maximum age-predicted HR, and/or volitional fatigue). Individuals were instructed to pedal until discomfort or fatigue prevented them from maintaining the required cadence. Pulmonary gas exchange was measured on a breath-by-breath basis (MetaMax 3B; Cortex Biophysik GmbH), with the gas analyzer calibrated before each session according to the manufacturer’s instructions. HR was recorded continuously throughout using short-range telemetry (Polar S810). V̇O_2_ max was recorded as the average oxygen uptake across the final 15 s before the termination of the test.

### Statistical Analyses

A linear mixed effects (LMEs) model was used to assess change in perfusion and CVR in a priori defined ROIs in R (R Core Team 2016) using the lme4 package (Bates et al. 2015), within-subjects factor: time (baseline, post1, post2, and post3) and hemisphere (left and right), covariates (PETCO2, HR, and mean arterial pressure [MAP]); *P*-values were calculated from degrees of freedom estimated using Satterthwaite’s method (Kuznetsova et al. 2017). Where a significant effect was found, post hoc pairwise comparisons of the postexercise timepoints with baseline were conducted, and a false discovery rate (FDR) of *q* = 0.05 was used to correct for multiple comparisons.

The reliability of CBF was assessed from the 2 baseline PASL sequences (*n* = 10, 5 males; 25.1 ± 1.3 years old) using the intraclass correlation coefficient (ICC; type A, absolute agreement definition). All data are expressed as mean ± standard error of the mean (SEM) unless stated.

## Results

### Exercise Intervention Checks

HR was significantly elevated throughout the exercise intervention compared to the upright rest period on the ergometer, with participants working at an average 57.2 ± 1.6% HRR. Average ratings of perceived exertion were 3.63 ± 0.20 in the legs and 3.19 ± 0.17 for breathing, associated with the verbal anchor “moderate’’ to “somewhat hard’’ exertion on the Borg 0–10 RPE scale. Average blood lactate levels were 2.97 ± 0.20 mmoL (resting levels = 0.83 ± 0.1 mmoL). On average, participants cycled at a workload of 104.0 ± 4.8 W, at a speed of 71.3 ± 2.6 revolutions per minute. Results across the time course of the intervention are shown in Supplementary Figure 1.

### Acute Exercise Effects on Gray Matter Perfusion

The ICC, calculated to assess reliability of perfusion at baseline, was between 0.45 and 0.8 for the various ROIs (see Supplementary Table 1) indicating fair to excellent agreement between the 2 baseline measures (Cicchetti 1994). A representative perfusion and arterial transit time map is shown in Supplementary Figure 2.

**Figure 2:**
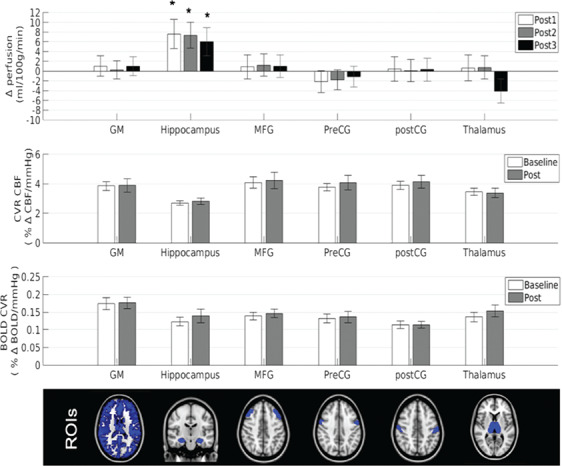
Resting perfusion and CVR from ASL-MRI following a single session of exercise. Top panel shows the change in resting perfusion from baseline with marginal means adjusted for PETCO_2_, MAP, and HR, error bars represent the change in standard error of the mean *P < 0.05 FDR-adjusted. Middle panels show CBF CVR and CVR BOLD values to hypercapnia obtained using a dual-echo ASL sequence; error bars represent the standard error of the mean. The ROI masks are shown in the bottom panel. GM means gray matter; MFG, middle frontal gyrus; preCG, precentral gyrus; postCG, postcentral gyrus.

Figure 2 shows the change in perfusion in the ROIs. Table 2 details the cardiorespiratory and cerebrovascular descriptive data and associated *F* statistics. Globally, there was no significant difference in gray matter perfusion following exercise compared to baseline, after accounting for changes in cardiorespiratory measures (*P* > 0.05). A main effect of exercise was observed in the hippocampus (*F*_3, 195.1_ = 3.24, *P* = 0.016). Post hoc analyses at the separate timepoints found a selective effect of exercise in the hippocampus for perfusion in the post-1 scan acquired 10–20 min after exercise cessation (*t*_201.8_ = 2.75, *P* = 0.011), with a 12.58% increase in hippocampal CBF compared to baseline. At the post-2 scan, hippocampal CBF remained significantly elevated (*t*_196.7_ = 2.39, *P* = 0.011, 10.16%), and similarly, at the post-3 scan, 55–65 min after exercise cessation, an exercise-induced elevation of hippocampal CBF was still observed (*t*_191.1_ = 2.25, *P* = 0.026, 10.00%), suggesting a single session of exercise induces a transient increase in regional CBF.

**Table 2:** Cardiorespiratory and cerebrovascular exercise

		Post-exercise scan			LME *F* statistic				
	Baseline	1	2	3	Exercise	Exercise*Hem	PETCO2	HR	MAP
Cardiorespiratory measures											
HR, beats/min	63.86 ± 2.47	75.12 ± 1.92	69.14 ± 1.93	66.38 ± 1.76	26.32***						
MAP, mmHg	86.03 ± 1.56	88.09 ± 1.90	86.90 ± 1.99	87.69 ± 1.77	2.48						
PETCO_2_, mmHg	36.76 ± 0.88	35.05 ± 0.94	35.18 ± 0.84	35.73 ± 0.84	4.11**						
Perfusion (ml/100 g/min) in ROIs										
Gray matter	49.06 ± 2.06	50.49 ± 2.01	49.75 ± 2.42	50.15 ± 2.48	0.05		0.02	0.56	0.45
Middle frontal gyrus	44.25 ± 2.72	45.51 ± 2.60	47.77 ± 6.24	52.03 ± 7.42	0.41		0.4	0.81	0.31
Postcentral gyrus	49.27 ± 2.79	51.48 ± 2.82	50.33 ± 3.31	49.15 ± 5.10	0.03		1.13	3.45	2.49
Precentral gyrus	53.07 ± 2.78	52.48 ± 2.42	51.59 ± 3.01	51.24 ± 4.84	0.38		0.93	2.51	1.1
Thalamus	43.39 ± 3.39	46.66 ± 3.18	44.91 ± 3.44	39.16 ± 4.11	1.96	0.08	0.05	0.06	0.45
Hippocampus	62.13 ± 3.18	71.80 ± 3.81	70.49 ± 3.31	71.31 ± 5.97	3.24*	0.28	0.001	4.41*	1.79

Means and standard error of the mean are shown, along with the *F* statistic from the LME analyses. For the thalamus and hippocampus, mean and standard error are averaged across hemisphere. For the perfusion measures, PETCO2, HR, and MAP were covariates in the analysis. ***P < 0.001, **P < 0.01, *P < 0.05.

There were no main effects of exercise in CBF in any of the other cortical and subcortical ROIs (all *P* > 0.05, see Table 2). Additional exploratory analyses found no effect of exercise on hippocampal perfusion from the post-1 to the post-2 scan (*t*_198.5_ = 0.17, *P* = 0.86) or from the post-2 scan to the post-3 scan (*t*_191.4_ = 0.39, *P* = 0.69). A model comparison found that the physiological covariates did not explain the data, with a significant increase in hippocampal CBF observed both with and without covariates (see supplementary information). Arterial transit times in the hippocampus were within the normal range (MacIntosh et al. 2010) and were not significantly altered by exercise (*F*_3,203.4_ = 0.729, *P* = 0.54; baseline = 633 ± 31.6 ms, post-1 = 626.4 ± 27.2 ms, post-2 = 629.71 ± 36.1 ms, post-3 = 626.6 ± 43.2 ms).

There was no significant interaction between hemisphere and exercise in any of the ROIs and at any timepoint (all *P* > 0.05). There was a main effect of hemisphere on CBF in the post-1, post-2, and post-3 scan in the hippocampus (15.2%, 12.7%, and 17.7% higher CBF in the left hippocampus, respectively, *P* < 0.01 FDR-adjusted) but not in the thalamus (all *P* > 0.05).

After covarying for age and sex, V02max was not related to the change in hippocampal CBF (*r* = −0.06 and 0.09 left and right hemisphere, respectively, *P* > 0.1) or baseline hippocampal CBF (*r* = 0.25 and −0.19 left and right hemisphere, respectively, *P* > 0.1).

### Acute Exercise Effects on Gray Matter CVR

Figure 2 shows the CVR response at baseline and post exercise. There was no significant effect of exercise on CBF CVR in gray matter (*P* = 0.99) or in any of the ROIs (all *P* > 0.05). Similarly, there was no effect of exercise on BOLD CVR in gray matter (*P* = 0.23) or in any of the ROIs (all *P* > 0.05). The degree of hypercapnia induced at baseline and postexercise did not differ (baseline = 7.68 ± 0.27; post = 7.72 ± 0.48; *t*_12_ = −0.076, *P* = 0.94) and HR, respiration rate, and MAP did not change with hypercapnia at baseline or postexercise (*P* > 0.05). Resting baseline perfusion was not correlated with baseline CBF CVR in any of the ROIs (all *P* > 0.05).

## Discussion

In this study, we aimed to determine the effect of a single 20-min session of moderate-intensity exercise on cerebral perfusion. Hypocapnia, which modulates CBF (Ainslie and Duffin 2009), was observed after exercise and was therefore accounted for in the statistical analyses, alongside blood pressure and HR. In line with the literature on chronic exercise effects, we found an increase in regional perfusion in the hippocampus up to 65 min after exercise, in the absence of a change in CVR using ASL MRI. The perfusion change was specific to the hippocampus, with no change observed in the thalamus, middle frontal gyrus, precentral gyrus, or postcentral gyrus.

Our data indicate that a single session of aerobic exercise selectively targets perfusion to the hippocampus. This result is particularly noteworthy given that the hippocampus has been the focus of exercise research due to its selective neurogenic capabilities in adulthood in humans (Eriksson et al. 1998) and that converging evidence in rodents and humans indicate that a hallmark effect of long-term aerobic exercise is to increase perfusion in the hippocampus (Pereira et al. 2007). This finding is specific to CBF, with no change in arterial transit time in the hippocampus. Furthermore, the absence of a corresponding localized change in hippocampal CVR suggests that immediate and transient changes in hippocampal perfusion after exercise are not driven by a mechanical vascular change and instead more likely represents an adaptive metabolic change due to the transient higher energy demands of hippocampal neurons. Previous studies have also suggested that increases in blood flow velocity observed during exercise may be caused by increased metabolism (Ide et al. 1999; Querido and Sheel 2007; Secher et al. 2008). Interestingly, a decrease in regional perfusion in the hippocampus has also been observed in athletes after 10 days of exercise cessation or detraining (Alfini et al. 2016). Thus, our findings support the notion that the regulation of resting hippocampal perfusion is responsive to short-term changes (increases or decreases) in exercise training.

It is not clear if the mechanisms for exercise-induced perfusion alterations are the same for acute transient effects and more stable chronic effects, despite the same hippocampal specificity. One plausible explanation for the observed perfusion change is the exercise-induced increase in catecholamine circulation. Experimental studies in both animal and humans show a transient increase in norepinephrine and dopamine release in several brain regions including the hippocampus during and following exercise (Meeusen and De Meirleir 1995; Dunn et al. 1996; Goekint et al. 2012; Skriver et al. 2014) although evidence remains equivocal (McMorris 2016). Dopamine works as a vasopressor and modulates local CBF (Krimer et al. 1998), thus cerebrovascular regulation may be affected by an exercise-induced alteration of dopaminergic transmission. However, given the rich perivascular dopaminergic terminals in the frontal cortex, it is not clear why effects were not observed in the cortical ROIs in this study. Future pharmacological studies are required to examine this hypothesis by directly manipulating dopaminergic systems during and/or following exercise. In contrast, a cerebral angiogenic response has been demonstrated following long-term exercise (Ding et al. 2006), with evidence of structural modifications to the cerebrovasculature and specifically changes in hippocampal cerebral blood volume observed after a long-term exercise intervention in mice and humans (Pereira et al. 2007). In humans, hippocampal blood volume increased after 12-weeks of exercise and correlated with cardiopulmonary and cognitive function (Pereira et al. 2007), whereas in mice, cerebral blood volume in the dentate gyrus correlated with neurogenesis. Despite this, the coupling between angiogenic processes and neurogenesis may not be consistent for all plasticity processes. For example, both memory gains produced by VEGF overexpression, and memory impairments produced by VEGF blockage, have been shown at early time points prior to new neurons becoming functional (Licht et al. 2011), suggesting that increased plasticity of mature neurons may also occur. The observed increase in CBF following an acute bout of exercise requires further investigation to determine if the duration and intensity is sufficient to induce angiogenic and/or neurogenic processes seen previously after longer periods.

A limitation of this study is the lack of a control group whom did not complete the intervention. Instead, we measured the reliability of our CBF measure at baseline and from this, are assured that our measure is stable across the time period in this study and robust to postural change. Future studies would benefit from including a comparative control group who are seated on the cycle ergometer for the intervention period but do not undergo exercise. In addition, future work would extend the postexercise scan period to examine how long the hippocampal CBF remains elevated for beyond the 65 min in this study.

## Conclusion

Overall, we demonstrate that acute exercise induces temporally and regionally specific changes in hippocampal perfusion and provides a framework for exploring the therapeutic potential of exercise in clinical and aged populations where long-term exercise-related functional gains have been observed. We show that a single session of aerobic exercise is sufficient to produce transient changes in hippocampal perfusion although further work is necessary to determine whether this represents the earliest stages of neural and/or vascular plasticity or a distinct transient mechanism. The selective responsiveness in hippocampal perfusion to exercise is particularly interesting in light of studies suggesting that the hippocampus is vulnerable to cognitive aging processes (Small et al. 2002, 2004) in humans (West et al. 1994; Small et al. 2002), primates (Gazzaley et al. 1996; Small et al. 2004), and rodents alike (Shetty and Turner 1999; Small et al. 2004).

## Funding

Wellcome Trust (200804/Z/16/Z); Waterloo Foundation.
